# PHLDA2 is a key oncogene-induced negative feedback inhibitor of EGFR/ErbB2 signaling via interference with AKT signaling

**DOI:** 10.18632/oncotarget.3674

**Published:** 2015-04-09

**Authors:** Xiaoqi Wang, Guangyuan Li, Sanjay Koul, Rieko Ohki, Matthew Maurer, Alain Borczuk, Balazs Halmos

**Affiliations:** ^1^ Division of Hematology/Oncology, Herbert Irving Comprehensive Cancer Center, Columbia University Medical Center, New York, NY, USA; ^2^ Radiobiology Division, National Cancer Center Research Institute, Tokyo, Japan; ^3^ Department of Pathology, Columbia University Medical Center, New York, NY, USA; ^4^ Department of Pathology, University Hospitals of Case Medical Center, Case Western Reserve University, Cleveland, OH, USA

**Keywords:** PHLDA2, EGFR, ErbB2, AKT

## Abstract

Pleckstrin homology-like domain family A member 2 (PHLDA2) is located within the tumor suppressor region of 11p15, and its expression is suppressed in several malignant tumor types. We recently identified PHLDA2 as a robustly induced, novel downstream target of oncogenic EGFR/ErbB2 signaling. In an immunohistochemical study, we find that PHLDA2 protein expression correlates positively with AKT activation in human lung cancers corroborating our data that PHLDA2 is induced upon oncogenic activation and might serve as a biomarker for AKT pathway activation. We show that PHLDA2 overexpression inhibits AKT phosphorylation while decreased PHLDA2 expression increases AKT activity. We further find that PHLDA2 competes with the PH domain of AKT for binding of membrane lipids, thereby directly inhibiting AKT translocation to the cellular membrane and subsequent activation. Indeed, PHLDA2 overexpression suppresses anchorage-independent cell growth and decreased PHLDA2 expression results in increased cell proliferation and reduced sensitivity to targeted agents of EGFR/ErbB2-driven cancers demonstrating functional relevance for this interaction. In summary, our studies demonstrate that PHLDA2 is strongly regulated by EGFR/ErbB2 signaling and inhibits cell proliferation via repressing AKT activation in lung cancers in a negative feedback loop. We highlight a novel action for PHLDA2 as a potential biomarker for AKT pathway activation.

## INTRODUCTION

The human epidermal growth factor receptor (HER) family of receptor tyrosine kinases (RTKs) is a well-established target for anticancer therapies. In particular, EGFR and ErbB2 are members of the HER family of receptor tyrosine kinases and are some of the best characterized oncogenes [1, 2]. EGFR-mutated lung adenocarcinomas and ErbB2-positive breast and other cancers (such as gastric and lung) are critically dependent on the constitutive activity of these pathways and therapeutic targeting leads to significant clinical benefits [3–6]. In particular, the small molecule EGFR TKI inhibitors, erlotinib and gefitinib show dramatic activity in EGFR-mutated lung adenocarcinomas and the anti-ErbB2 antibody, trastuzumab and the dual EGFR/ErbB2 inhibitor, lapatinib have proven benefits in ErbB2-amplified malignancies [3, 7–11]. The overall benefit of these drugs still is limited due to the development of acquired resistance developing through secondary mutations of EGFR or alternative pathway activation in the great majority of cases leading to reactivation of downstream pathways despite ongoing drug inhibition [12–15]. EGFR/ErbB2 activation results principally in enhanced signaling through the MAP kinase pathway and the PI3K/AKT pathway, leading to stimulation of cellular growth and progression, proliferation, blockade of apoptosis, angiogenesis, and invasion [16–18]. Therapeutic success in particular depends on the ability of inhibiting the AKT pathway leading to Bim downregulation and induction of apoptosis [19]. Better understanding of downstream pathway modulation is anticipated thereby to possibly allow novel opportunities for intervention.

In order to arrive at a comprehensive view of downstream signaling changes, we previously completed several transcriptional profiling studies identifying key groups of early EGFR and ErbB2 oncogenic pathway target genes. For example, we previously identified that the expression of the dual specificity phosphatase, DUSP6 is strongly regulated by ERK signaling and that DUSP6 exerts antitumor effects via robust negative feedback regulation [20]. In the present study, we focus further on a novel downstream target of EGFR and ErbB2 signaling, PHLDA2, identified repeatedly as an early target in our transcriptional profiling studies. PHLDA2, i.e. pleckstrin homology-like domain family A member 2, is the first apoptosis-related gene that was shown to be imprinted and expressed solely from the maternal allele in normal development [21, 22]. The function of PHLDA2 in cancers is still largely unclear albeit it is one of several genes in the imprinted gene domain of 11p15.5 which is considered to be an important tumor suppressor gene region [23]. Alterations in this region are frequent in different tumors, including lung and breast cancers suggestive of putative tumor suppressor functions [24–27]. PHLDA2 shares a pleckstrin homology domain (PH domain) with PHLDA1 and PHLDA3. PHLDA3 is a PH-domain only protein and functions as a unique AKT inhibitor through the depletion of membrane-bound phosphatidyl inositols [28]. The limited published studies do suggest that PHLDA2 is also capable of binding phosphatidyl inositol signaling intermediates and thereby might directly modulate AKT signaling [22]. As our transcriptional profiling studies consistently found PHLDA2 as an immediate downstream target of EGFR/ErbB2 signaling, we speculated that PHLDA2 might have key inhibitory functions in EGFR and ErbB2-driven cancer cells and a better understanding of its functions might help define novel therapeutic options, in particular through yielding novel ways of interfering with phosphatidyl inositol-mediated activation of oncogenic AKT-driven pathways.

## RESULTS

### PHLDA2 is down-regulated by inhibition of oncogenic EGFR/ErbB2 signaling

**Figure 1:**
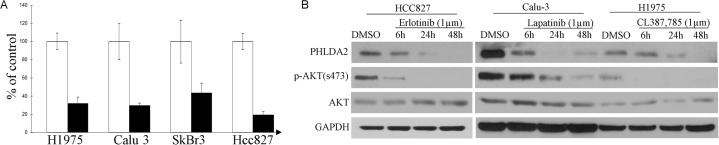
PHLDA2 is immediately regulated by ErbB2 inhibition **A.** Quantitative RT-PCR analysis of PHLDA2 regulation by CL387,785, lapatinib and Erlotinib treatment, respectively for 6 hours in H1975, Calu-3, SkBr3 and HCC827 cells. Fold induction relative to 0 hour control was plotted after normalization by GAPDH. **B.** Western Blot analysis of PHLDA2 expression treated with Erlotinib, lapatinib and CL387, 785 for 6, 24 and 48 hours in HCC827, Calu-3 and H1975 Cells.

Previously, we have shown that EGFR inhibition in EGFR-mutated lung adenocarcinoma cells could induce a very early and significant change of transcription in a small and representative group of key downstream effectors of oncogenic signaling, including important pathway components such as Cyclin D1, AP-1, dual specificity phosphatases etc [20, 29]. In further microarray studies of other EGFR and ErbB2-driven cancer cells (Table 1), a largely overlapping group of genes was consistently noted to be regulated. As PHLDA2 is repetitively included in this group, we then decided to focus on this novel downstream target gene that previously was not noted to be involved in EGFR/ErbB2-driven pathways. Real-time quantitative PCR analysis showed that expression of PHLDA2 was repressed to 43.5 ± 10.6% (mean ± SD) of lapatinib-treated as compared to DMSO-treated control cells in the ErbB2-positive SkBr3 breast cancer cell line, 31.7 ± 7.2% (mean ± SD) of lapatinib-treated versus DMSO-treated control cells in ErbB2-amplified Calu-3 lung adenocarcinoma cells, 19.2 ± 3.83% of Erlotinib treated versus DMSO-treated control cell in EGFR-mutated HCC827 and 29.4 ± 2.9% of CL-387, 785-treated versus DMSO-treated control cells in EGFR-mutated H1975 lung adenocarcinoma cells (Figure 1A). In addition, Western blotting analysis showed that PHLDA2 was down-regulated significantly as early as 6 hours upon EGFR or ErbB2 inhibition on the protein level as well (Figure 1B). These results suggest that PHLDA2 is immediately and significantly down-regulated by suppression of oncogenic EGFR/ErbB2 signaling on both the mRNA and protein levels.

**Table 1:** Transcriptional profiling study of PHLDA2 regulation with TKI treatment in breast and lung cancer cell lines

Cell line	Tissue	EGFR/ErbB2	Drug treatment	PHLDA2 regulation with TKIs treatment
HCC827	Lung	EGFR DelE746-A750, amplification	Erlotinib	Down regulation (q-PCR, Western blot)
H1975	Lung	EGFRL858R+ T790M	CL387, 785	Down regulation (Transcriptional profiling study, q-PCR, Western Blot)
Calu-3	Lung	ErbB2 amplification	Lapatinib	Down regulation (q-PCR, Western Blot)
SkBr3	Breast	ErbB2 positive	Lapatinib	Down regulation (Transcriptional profiling study, q-PCR, Western Blot)

### PHLDA2 protein expression correlates positively with AKT activation in lung cancer cell lines and primary human lung tumors

**Figure 2:**
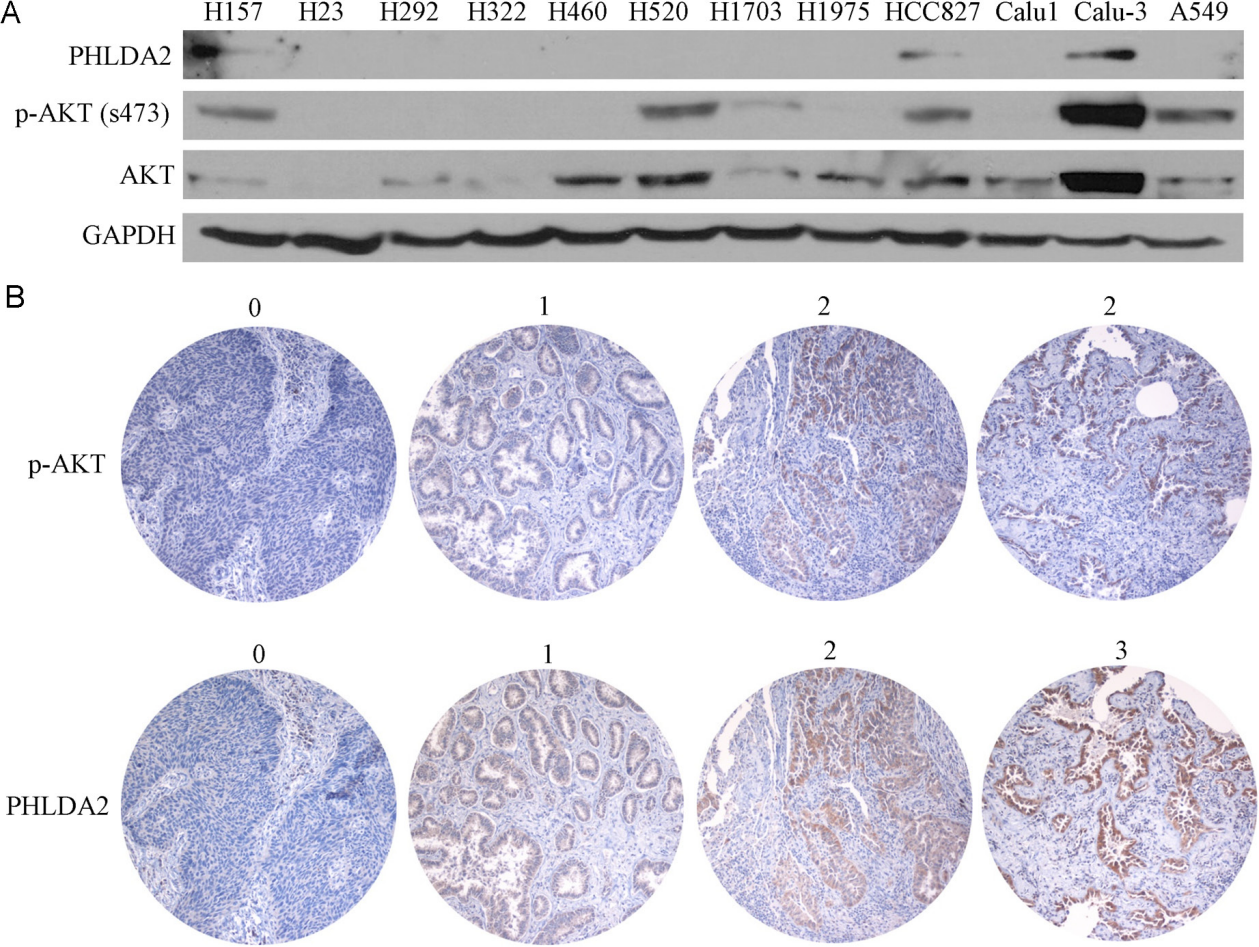
PHLDA2 protein expression correlates positively with AKT activation in lung cancer cell lines and human lung cancer tissue microarray **A.** Western blotting of PHLDA2, p-AKT and AKT in 12 lung cancer cell lines. **B.** NSCLC tumor sessions were subjected to immunohistochemistry to detect PHLDA2 and activated AKT. 0 (no expression), 1 (weak expression), 2 (moderate expression) and 3 (strong expression).

We next investigated cellular expression of PHLDA2 by immunoblot in 12 NSCLC cell lines, correlated to p-AKT expression to assess AKT-activation (Figure 2A). We observed PHLDA2 expression in 3 lung cancer cell lines with high p-AKT expression and 100% of cell lines lacking p-AKT expression had absent expression of PHLDA2 as well suggestive of a correlation. As 3 lung cancer cell lines with detectable p-AKT expression on the other hand had no PHLDA2 expression, expression in some cases appears to be regulated by a combination of factors other than p-AKT activation as well.

**Table 2:** Statistical analysis of the correlation between PHLDA2 and p-AKT levels for lung cancer tissue microarray (*p* = 0.0002)

	p-AKT		
PHLDA2	no staining	mild staining	moderate staining
no staining	22(38.6%)	6(12.8%)	1(7.7%)
mild staining	25(43.9%)	25(53.2%)	5(38.5%)
moderate staining	9(15.8%)	15(31.9%)	6(46.2%)
strong staining	1(1.7%)	1(2.1%)	1(7.7%)

In order to better understand the correlation of AKT activation and PHLDA2 expression, we then developed an IHC protocol for assessing the correlation of PHLDA2 and AKT activation and scoring was performed by an expert lung pathologist (Figure 2B). The distribution of PHLDA2 staining of the 117 interpretable tumors in the tissue microarray (TMA) is presented in Table 2: no staining (*n* = 29; 24.8%); mild staining (*n* = 55; 47.0%); moderate staining (*n* = 30; 25.6%) and strong staining (*n* = 3; 2.6%). The distribution of p-AKT staining of the 117 interpretable tumors in the tissue microarray (TMA) was as follows: no staining (*n* = 57; 48.7%); mild staining (*n* = 47; 40.2%) and moderate staining (*n* = 13; 11.1%). There was a strong correlation noted between PHLDA2 and p-AKT expression (correlation coefficient = 0.336, *p* = 0.0002). Only 7.7% (1/13) specimens with moderate p-AKT staining showed no PHLDA2 staining while 38.6% (22/57) specimens with no p-AKT staining showed no PHLDA2 staining. We also observed that 75.9% (22/29) specimens without PHLDA2 staining had no p-AKT staining. We further analyzed the correlation of PHLDA2 and ERK activation and found no significant correlation (*p* > 0.05) suggestive of a specific link with AKT signaling activity. We also observed a significant correlation of PHLDA2 expression with histological subtype and a strong trend towards correlation of PHLDA2 expression with EGFR mutational status (Table 3). Only 6.7% (1/15) specimens with EGFR mutation showed no PHLDA2 staining as compared with 25% (10/40) EGFR wild type specimens had absent PHLDA2 staining. The correlation of PHLDA2 expression with both p-AKT and EGFR mutation corroborates that PHLDA2 expression is indeed activated by oncogenic EGFR/AKT signaling in primary lung tumors as well.

**Table 3:** Statistical analysis of the correlation between PHLDA2 and pathological subtypes for lung cancer tissue microarray (*p* = 0.0179)

Pathological subtype	PHLDA2	
	no staining	staining
adenocarcinoma	12(16%)	63(84%)
Other types	16(38.1%)	26(61.9%)

### PHLDA2 represses AKT under both inducible and basal conditions

**Figure 3:**
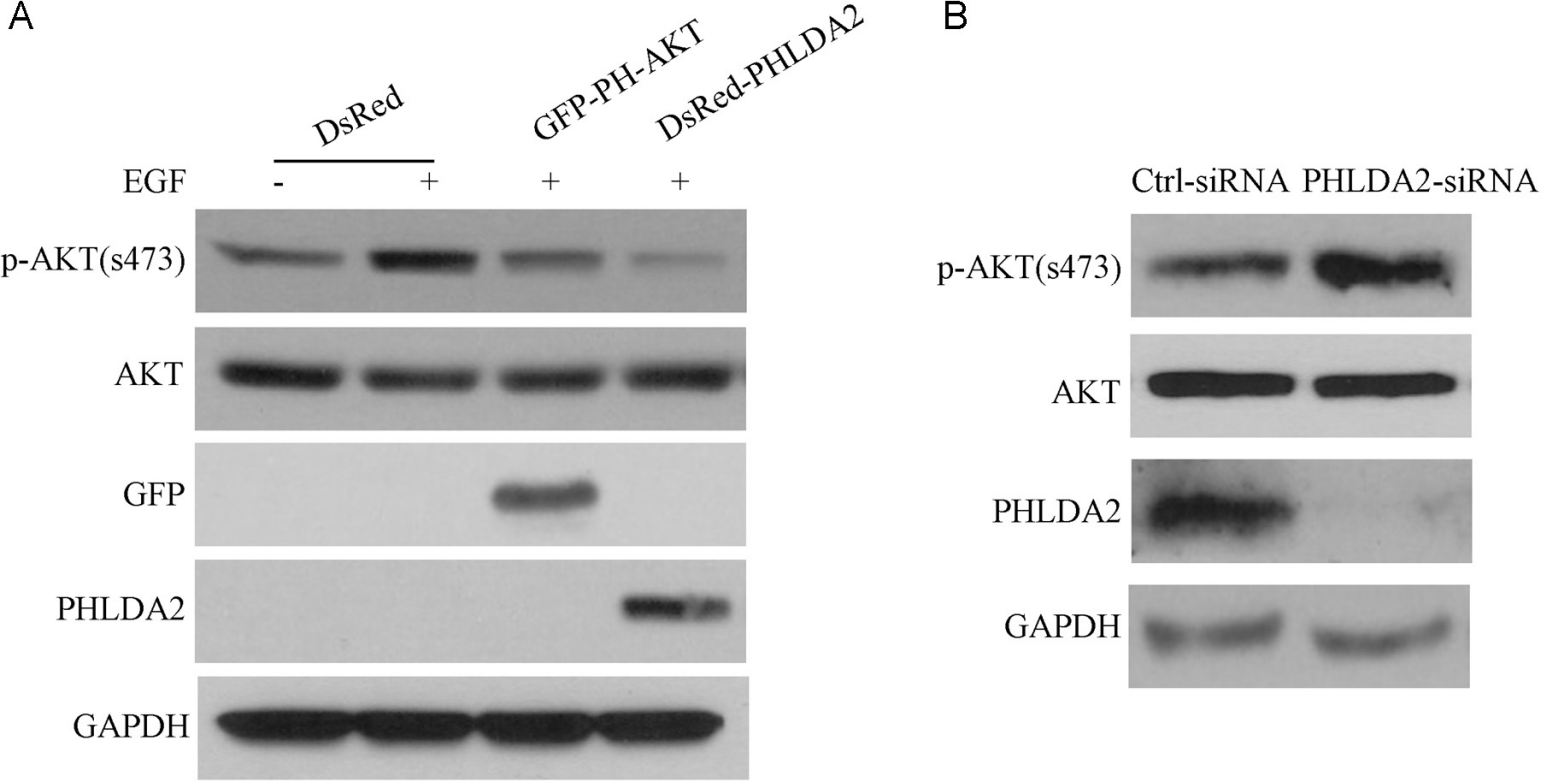
PHLDA2 inhibits AKT activation **A.** COS7 cells were transfected with the indicated fusion proteins for 24 hr and subsequently stimulated with EGF for 15 min. AKT activity after EGF treatment was analyzed by western blotting. **B.** Control or PHLDA2-targeting siRNAs were introduced into Calu-3 cells. Cells were harvested 72 hr post-transfection and analyzed by western blotting.

The closest paralogue of PHLDA2 is PHLDA3, another small PH domain protein which has been reported to inhibit AKT activation through interfering with AKT binding to membrane lipids. To investigate the biochemical function of PHLDA2 on AKT activity in NSCLC, we generated PHLDA2-overexpressing cells and control cells by transfecting cells with the DsRed-PHLDA2 expression vector and the empty vector DsRed. We also generated PHLDA2-knockdown cells and corresponding control cells by transfecting cells with siRNA targeting PHLDA2 and control scramble siRNA. PHLDA2 protein levels in PHLDA2 overexpressing and PHLDA2-knockdown cells were confirmed by Western blotting. As shown in Figure 3A, PHLDA2-overexpressing cells showed significantly upregulated expression of PHLDA2 protein as analyzed by Western Blot in COS7 cells compared with control cells. AKT activation was investigated 24 h post-transfection following 15 min of EGF stimulation. EGF significantly induced AKT phophorylation in the DsRed empty vector group but not in GFP-PH-AKT and DsRed-PHLDA2 group. As expected, GFP-PH-AKT, which was set as a positive control in this experiment, repressed AKT activity by inhibition of the AKT pathway. Importantly, we found that PHLDA2 overexpression similarly resulted in diminished AKT phosphorylation at Ser473, which is essential for AKT activity. In addition, we analyzed the effect of PHLDA2 knockdown under basal conditions in ErbB2-positive Calu-3 cells (Figure 3B). PHLDA2 siRNA-treated cells showed significantly decreased expression of PHLDA2 protein in Calu-3 cells compared with control cells. In PHLDA2 knock-down cells, AKT activity as assessed by phosphorylation at Ser473 was increased. These data in the aggregate demonstrate that PHLDA2 represses AKT activity analogous to GFP-PH-AKT suggesting that PHLDA2 functions as an AKT antagonist.

### PHLDA2 interferes with the translocation of AKT to the plasma membrane

**Figure 4:**
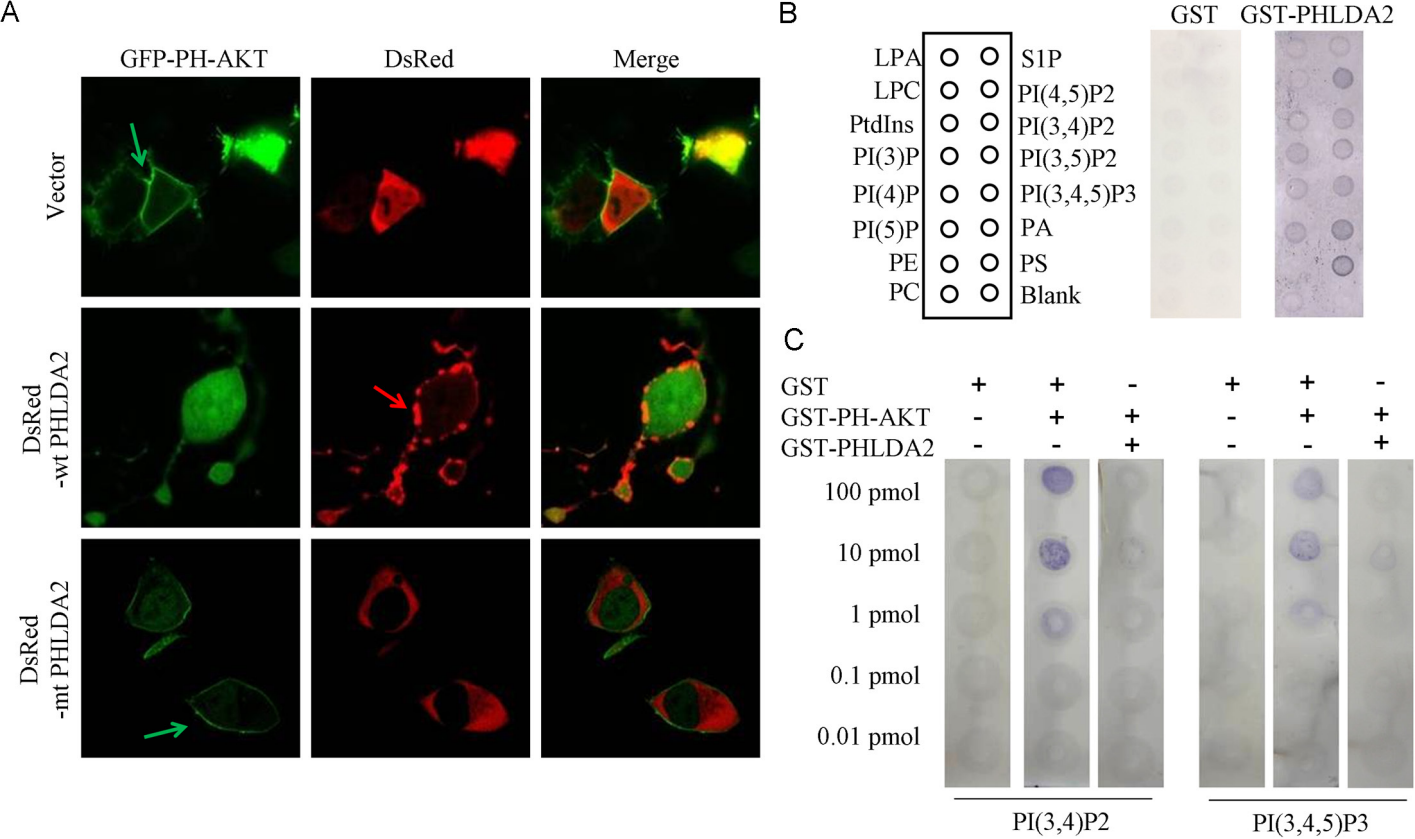
PHLDA2 interferes with AKT translocation to the plasma membrance and binding to PIP2 and PIP3 **A.** PHLDA2 interferes with AKT to localize to the cell membrane. 293T cells were transfected with GFP-PH-AKT together with DsRed, DsRed-WT PHLDA2 or DsRed-mtPHLDA2 and subcellular localization was analyzed. Green and red arrows indicate AKT and PHLDA2 localized at the plasma membrane respectively. **B.** Binding of GST-PHLDA2 to PIP was assessed by protein-overlay assay. PIP strips were spotted with 15 different biologically active lipids at 100 pmol per spot. Bound proteins were detected with anti-GST antibody and detected with K-TMBP substrate. **C.** PHLDA2 inhibits PH-AKT binding to PIP2 and PIP3. Binding of GST-PH-AKT to immobilized PIP was assessed by protein-lipid overlay assay. Nitrocellulose membranes spotted with serially diluted PIP2 and PIP3 were incubated with the indicated proteins. While GST did not interfere with AKT binding to PIP, PHLDA2 significantly interfered. Bound AKT was detected with anti-AKT PH domain antibody.

When PI(3,4,5)P3 and PI(3,4)P2 are generated by PI3K upon growth stimulation, AKT translocates from the cytoplasm to the plasma membrane by binding to these PIP isoforms through its PH domain, which specifically binds to PI(3,4,5)P3 or PI(3,4)P2. Such PIP binding is required for phosphorylation and activation of AKT. Since PHLDA2 inhibits AKT activity under both induced and basal conditions, we analyzed whether PHLDA2 expression inhibits AKT translocation to the plasma membrane. We assessed the effect of PHLDA2 expression on AKT localization in 293T cells with constitutively active PI3K activity thereby serving as an ideal model system. We co-expressed DsRed or DsRed-WT PHLDA2 together with GFP-PH-AKT in 293T cells. GFP-PH-AKT has been shown to mimic AKT translocation upon PI3K pathway activation and thereby it serves as a marker for PI3K pathway activity. 293T cells expressing DsRed resulted in GFP-PH-AKT translocation to the plasma membrane as expected, whereas in cells expressing DsRed-wt PHLDA2, GFP-PH-AKT translocation was drastically inhibited. Moreover, such inhibition was not observed in cells expressing DsRed-mt PHLDA2, a PHLDA2 mutant with a defective PH domain in which we deleted two key series of amino acids impairing the ability of the PHLDA2 PH domain to bind with high affinity to PtdIns(3,4,5)P3 and PtdIns(3,4)P2 (Figure 4A). Taken together, the results show that PHLDA2 impedes AKT translocation to the cellular membrane and subsequent activation in a manner dependent on its PH domain function.

### PHLDA2 directly interferes with AKT binding to PIP2 and PIP3

Because PHLDA2 expression led to the inhibition of AKT translocation to the plasma membrane and subsequent phosphorylation and activation, we next analyzed the mechanism by which PHLDA2 inactivates AKT. To directly assess the PIP binding properties of PHLDA2, we carried out protein-lipid overlay experiments, using GST fusion proteins containing PHLDA2 to probe PIP strips which have been spotted with 15 different biologically active lipids. As shown in Figure 4B, results were consistent with their expected binding specificities, GST-PHLDA2 was capable of binding to most PIPs, including PtdIns(3,4)P2 and PtdIns(3,4,5)P3 as well as several of the PtdIns monophosphates [30], while GST alone did not bind any of the PIP isoforms to an appreciable degree. We then performed an *in vitro* competitive protein-lipid overlay assay to see if PHLDA2 directly interferes with AKT binding to PIPs utilizing PI(3,4,5)P_3_ and PI(3,4)P_2_ as the primary test ligands. PHLDA2 and AKT were mixed in a molar ratio of 1:1 and reacted with PI(3,4,5)P3 and PI(3,4)P2 to test whether PHLDA2 competitively interferes with AKT binding to PIP. As shown in Figure 4C, AKT binding to PI(3,4,5)P3 and PI(3,4)P2 was significantly inhibited by PHLDA2 but not when AKT was mixed with control GST. Collectively, these results demonstrate that PHLDA2 represses AKT activity by competitively binding PI(3,4,5)P3 and PI(3,4)P2.

### PHLDA2 inhibits anchorage dependent and independent cell growth and significantly enhances treatment sensitivity in EGFR/ErbB2-driven cancer cells

**Figure 5:**
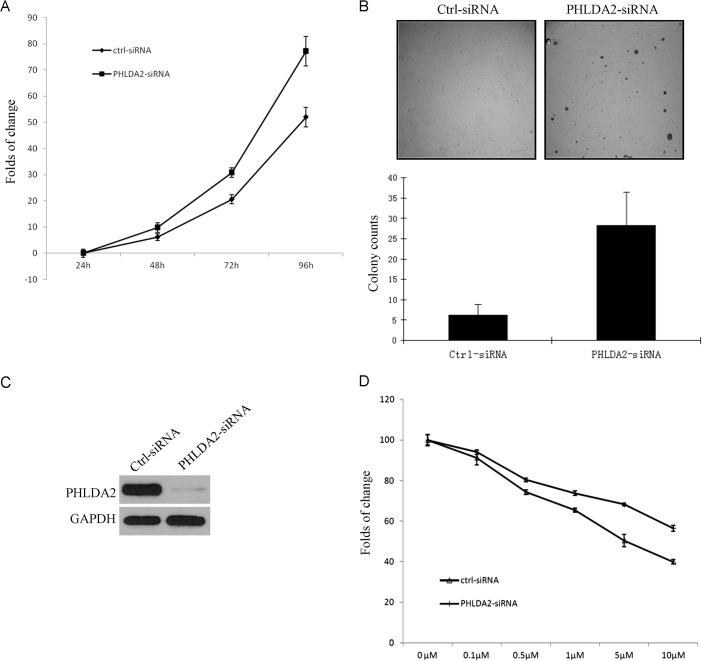
PHLDA2 suppresses lung cancer cell growth and increases lapatinib drug sensitivity **A.** MTS assay shows that the growth of HCC827 cells transfected with PHLDA2 siRNA was significantly increased at day 3 compared with cells transfected with control siRNA. **B.** PHLDA2 siRNA cells and control siRNA cells were plated in soft agar and cultured for 4 weeks. Colonies were counted from three plates and the mean numbers of colonies ± SD are shown. Images and Bar graph show significantly larger and more colonies in PHLDA2 knockdown cells. **C.** PHLDA2 knock-down efficiency as shown by Western blotting for PHLDA2 expression at 48 hours. **D.** MTS assay shows that Calu-3 cells transfected with PHLDA2 siRNA are significantly less sensitive to lapatinib treatment as compared to cells transfected with control siRNA.

Since PHLDA2 mediates AKT inhibition, we further analyzed the functional role of PHLDA2 in tumor growth of NSCLC cells. We first examined the effects of siRNA-mediated silencing of PHLDA2 expression on *in vitro* cell growth in EGFR-mutated HCC827 cells. PHLDA2 siRNA was successfully transfected into the cells and reduced PHLDA2 expression leading to significant cell proliferation (Figure 5A). We then analyzed the effect of PHLDA2 knockdown on colony formation efficiency. PHLDA2 expression under basal conditions was analyzed and was almost undetectable in PHLDA2-siRNA cells (Figure 5C). As shown in Figure 5B, PHLDA2 knockdown significantly increased the number of colonies and the individual colony sizes were notably larger than those of the controls in soft agar, suggesting that PHLDA2 expression decreases the autonomous growth potential of NSCLC cells. These results indicate that PHLDA2 inhibits anchorage-dependent and independent cell growth and survival in NSCLC cells, suggesting that PHLDA2-modulated pathways could be novel therapeutic targets for NSCLC. To examine whether expression of PHLDA2 enhances sensitivity of NSCLC cells to targeted agents, MTS cell proliferation assay was used to assess the role of PHLDA2 in lapatinib sensitivity of ErbB2-positive cancer cells. Calu-3 cells were transfected with PHLDA2 siRNA and control siRNA for 48 hours and then cultured in the presence of lapatinib and allowed to continue to grow for 3 days, followed by MTS staining. The results showed that Calu-3 cells following PHLDA2 knockdown were significantly less sensitive to lapatinib than the control siRNA group (Figure 5D). These results indicated that constitutive PHLDA2 expression can clearly enhance the sensitivity of ErbB2-positive cancer cells to ErbB2-targeting agents.

## DISCUSSION

EGFR and ErbB2-targeting for molecularly defined cancers, such as EGFR-mutated lung adenocarcinomas and ErbB2-amplified breast and other cancers have demonstrated great promise but the emergence of acquired resistance limits long-term efficacy and better understanding of resistance mechanisms as well as identification of key downstream pathway activities and dependencies are pivotal for the design of novel strategies to prevent and combat resistance [13]. EGFR dimerization results in autophosphorylation, kinase activation and subsequent activation of two major pathways, including RAS/RAF/MEK/ERK1/2 and PI3K/AKT pathways [31, 32]. In previous studies, we defined the comprehensive downstream changes upon inhibition of oncogenic EGFR/ErbB2 in EGFR/ErbB2-addicted tumor models and identified a very select number of genes altered as early as 6 hours following EGFR/ErbB2 blockade [29]. We confirmed and functionally analyzed several of these genes and many are known to participate in key aspects of oncogenic signaling modulation. For example, ERK1/2 signaling is negatively regulated by a family of dual-specific MAPK phosphatases, known as DUSPs [20]. DUSP1 and DUSP4 function to terminate ERK signaling in nucleus whereas DUSP6 inhibits ERK activation in the cytoplasm [14, 33]. We and others found that DUSPs are some of the most robustly regulated downstream targets of EGFR/ErbB2 signaling and indeed both DUSP4 and DUSP6 independently serve key modulatory functions and might be candidate tumor suppressor genes [20]. In the present study, we focused on the strongly regulated novel gene by EGFR/ErbB2 blockade, PHLDA2 as it has not been investigated before as a target of oncogenic pathways in cancers. Through quantitative RT-PCR and Western blot assays, we first verified PHLDA2 down regulation by EGFR/ErbB2 blockade in multiple models and confirmed PHLDA2 as a downstream target of EGFR/ErbB2. Next, we demonstrated that in human lung cancer cell lines and primary lung cancer specimens PHLDA2 expression correlates with p-AKT suggestive of a direct regulation. Further, we pursued studies to understand the mechanistic function of PHLDA2 and demonstrate that it can serve as a novel negative modulator of AKT by inhibiting AKT translocation to the cellular membrane and thereby AKT activation. Lastly, we demonstrate that PHLDA2 expression has functional impact in EGFR/ErbB2-driven cancer cells and can modulate the efficacy of molecularly targeted therapy.

Recently, genomic imprinting, an epigenetic form of gene regulation that results in the expression of only one parental allele, was shown to not only play an important role in embryogenesis and behavioral development but also to contribute to carcinogenesis [34]. PHLDA2, one of the first identified maternally expressed genes imprinted in placenta and most fetal tissues, has been implicated in early growth and development [22]. Increased PHLDA2 expression is also associated with placenta growth restriction and low birth weight [35]. However, only a few studies have reported on the relationship between PHLDA2 and human cancers, including the loss of PHLDA2 expression in complete hydatidiform moles, Wilms’ tumors, brain cancer and osteosarcoma [21, 36–38]. Moreover, besides the study on the potential tumor suppressor role with negative growth regulation in osteosarcoma [39], those studies generally focused on the expression level and the imprinting status of PHLDA2 in those cancers and no reports on the expression pattern of PHLDA2, its functional role and involved mechanisms of regulation in non-small cell lung cancer have been published.

A major downstream signaling route of EGFR is via the Ras-Raf-MAPK pathway. Activation of Ras initiates a multistep phosphorylation cascade that leads to the activation of MAPKs, ERK1 and ERK2, which ultimately regulate transcription of molecules involved in cell proliferation [11]. Another important target in EGFR signalling is phosphatidylinositol 3-kinase (PI3K) and the downstream protein-serine/threonine kinase AKT which transduces molecular signals triggering crucial steps for cell growth and survival [32]. Since PHLDA2 is a downstream target of EGFR/ErbB2, we analyzed PHLDA2, p-Erk and p-AKT expression in multiple lung cancer cell lines and a tissue microarray prepared with 117 well annotated primary lung cancer samples. Interestingly, we found PHLDA2 expression in 3 lung cancer cell lines with high p-AKT expression and 100% of cell lines without p-AKT expression also lacked expression of PHLDA2. In the tissue microarray, we found that PHLDA2 expression was significantly associated with AKT activation (*p* = 0.0002) but not with ERK activation (*p* > 0.05). We also found a strong trend of PHLDA2 expression correlating with the presence of EGFR mutations. These data confirmed that PHLDA2 protein expression correlates positively with AKT activation in lung cancer cell lines and human lung cancer tissue samples. Further research will be necessary to better identify the actual mechanism of regulation of PHLDA2 expression as well as larger scale tissue-based studies will be needed to better address if PHLDA2 expression could indeed serve as a useful biomarker for AKT pathway activation. Taken together, it appears that PHLDA2 expression is strongly regulated by EGFR/ErbB2 blockade and we speculate that it might possibly serve as an AKT inhibitor via negative feedback regulation and fulfill a significant function in oncogenic signaling modulation.

A recent key study showed that PHLDA3, a homologue of PHLDA2, is in fact a P53 regulated repressor of AKT and the loss of the PHLDA3 genomic locus is frequently observed in primary lung cancers, suggesting a role of PHLDA3 in tumor suppression [28]. PHLDA3 is expressed in multiple fetal and adult tissues, while the PHLDA2 gene is expressed in the placenta and fetal liver, indicating that these two genes have non-overlapping functions in development [22, 28]. Since we found that PHLDA2 expression correlates positively with AKT activation in lung cancer we then analyzed the effect of PHLDA2 on AKT activity by modulating PHLDA2 expression in lung cancer cells via overexpression and knockdown studies. PHLDA2 overexpression indeed inhibits AKT phosphorylation while PHLDA2 knockdown increases AKT phosphorylation with both sets of experiments confirming that PHLDA2 can functionally impair AKT activation.

AKT activation is regulated by a dual mechanism that requires both translocation to the plasma membrane and phosphorylation at Thr308 and Ser473 [40]. Mitogenic signals by receptor tyrosine kinases are first transmitted to phosphatidylinositol 3-kinase (PI3K), leading to production of the second messenger, phosphatidylinositol (3,4,5)-trisphosphate (PIP_3_) [41]. PIP_3_ generation on the inner aspect of the plasma membrane leads to translocation and activation of AKT [42]. AKT is recruited to the plasma membrane through direct interaction with its PH domain which functions as a lipid-binding module [40]. We set out to study how PHLDA2 interferes with this AKT-membrane phospholipid interface. First, we assessed the subcellular localization of AKT and PHLDA2 in 293T cells which have a constitutively active PI3K/AKT pathway and found that PHLDA2 interferes with AKT translocation to the plasma membrane. Since PHLDA2 expression leads to the inhibition of AKT translocation to the plasma membrane and subsequent phophorylation and activation, we next analyzed the mechanism by which PHLDA2 inactivates AKT. Both PHLDA2 and PHLDA3 are mostly composed of the PH domain and both bind to PIPs with broad specificity. PHLDA3 was previously shown to function as a unique AKT inhibitor through the depletion of membrane-bound phosphatidyl inositols [28], however, the physiological function of PHLDA2 in lung cancer has not been defined. Results by an *in vitro* protein-lipid overlay assay demonstrated that PHLDA2 interferes with AKT binding to PI(3,4,5)P3 and PI(3,4)P2 directly. Therefore, PHLDA2 inactivates AKT through the depletion of membrane-bound PIPs. Identification of this novel mechanism for modulation of AKT pathway activation should serve as the foundation for novel AKT inhibitor discovery, for example peptidomimetic inhibitors mimicking PHLDA2 function.

As an important signaling pathway downstream of EGFR, AKT is associated with tumor cell survival, proliferation, and invasiveness [40]. The activation of AKT is also one of the most frequent alterations observed in human cancer and tumor cells [43]. Tumor cells that have constitutively active AKT may depend on AKT for survival [44]. Since we found that PHLDA2 inhibits AKT activation through interfering with AKT translocation to the plasma membrane and further showed that this is mediated by direct competition with AKT to bind membrane lipids, we next examined whether PHLDA2 acts as a functional tumor suppressor in lung cancer. We knocked down PHLDA2 in ErbB2-positive NSCLC cells and demonstrated that PHLDA2 knockdown increased NSCLC cell growth and reduced sensitivity of the NSCLC cells to the ErbB2 tyrosine kinase inhibitor, lapatinib, consistent with its putative function as an AKT pathway inhibitor and suggestive of a candidate tumor suppressor function.

In conclusion, we show PHLDA2 regulation by EGFR/ErbB2 signaling, demonstrate a correlation between PHLDA2 expression, AKT pathway activation and EGFR mutational status and show that PHLDA2 has key negative feedback inhibitory functions in EGFR/ErbB2-driven cancer cells. Better understanding of PHLDA2′s regulation and its interactions with membrane PIPs/inhibition of AKT activation can yield novel therapeutic options for the treatment of patients with EGFR/ErbB2-driven cancers.

## MATERIALS AND METHODS

### Reagents

EGF was purchased from Sigma-Aldrich (St. Louis, MO). CL387, 785 was purchased from Calbiochem (Billerica, MA), lapatinib was purchased from SELLECK Chemicals (Houston, TX). Drugs were dissolved in DMSO to give a 10 mmol/L stock solution and the final DMSO concentration in all experiments was <0.1%. PIP strips and PIP lipids were purchased from Echelon Research Laboratories (Salt Lake City, UT).

### Cell culture

Twelve NSCLC Cell lines were used: H157, H23, H292, H322, H460, H520, H1703, H1975, HCC827, Calu-1, Calu-3 and A549. Cells were maintained in RPMI 1640 medium supplemented with 10% FBS. One breast cancer cell line, SkBr3 was maintained in McCoy's medium supplemented with 10% FBS. 293T cells were maintained in DMEM medium supplemented with 10% FBS. All cells were purchased from ATCC and grown at 37°C in a humidified atmosphere with 5% CO_2_ and were in the logarithmic growth phase at the initiation of all experiments.

### Real-time quantitative reverse transcription-PCR

Total RNA from triplicate samples was isolated by using the RNAeasy mini kit (Qiagen Sciences Inc, Germantown, MD). cDNA was synthesized with M-MLV reverse transcriptase (SuperScipt™ III reverse transcriptase, Invitrogen, Grand Island, NY) with the use of oligo(dT) primers. All samples were analyzed by Roche LightCycler using Syber green probes (Roche Applied Science, Indianapolis, IN). Real-time quantitative PCR primers for PHLDA2 were: forward primer 5′-CGACAGCCTCTTCCAGCTAT-3′ and reverse primer 5′-ATTCATTCAAAGCCGGTTCC-3′. Levels of GAPDH expression were used as internal references to normalize input cDNA. Real-time quantitative PCR primers for GAPDH were: forward primer 5′-GCGGGGCTCTCCAGAACATCAT-3′ and reverse primer 5′-CCAGCCCCAGCGTCAAAGGTG-3′ Ratios of level of each gene to GAPDH were then calculated.

### Immunoblotting

Whole cell lysates were subjected to protein quantification and analyzed by Western blotting. To detect PHLDA2 and p-AKT, 50 μg of whole cell lysates were loaded. Anti-PHLDA2 monoclonal antibody was obtained from Abcam. Anti-AKT rabbit polyclonal antibody, anti-phospho-AKT (S473) rabbit polyclonal antibody and anti-GAPDH antibody were purchased from Cell Signaling Technology. Anti-GST mouse monoclonal antibody (clone GST-2) was obtained from Sigma and anti-AKT/PKB PH domain mouse monoclonal antibody from Upstate.

### Immunohistochemical studies

The expression of PHLDA2, p-AKT and p-ERK in high-density tissue microarrays (TMA) were detected by a standard biotin-avidin-complex-based immunohistochemical method with the use of a monoclonal mouse antibody against PHLDA2 (Cat. # ab58379, Abcam Inc, Cambridge, UK), a rabbit antibody against p-AKT (Cat. #4060 Cell signaling, USA) and a rabbit antibody against p-ERK (Cat. # 9102 Cell signaling, USA) at a 1:50 dilution. The TMA was generated in the Department of Pathology, Columbia University containing FFPE tissue sections of 117 annotated primary non-small cell lung cancer tissue sections and 11 normal lung samples to serve as controls. TMA slides were deparaffinized and rehydrated and incubated with 0.6% hydrogen peroxide in methanol. Target retrieval solution, pH 9.0 (Dako, Carpinteria, CA) was used for antigen retrieval. The slides were incubated with primary antibodies at 1:50 dilution for 30 minutes at room temperature followed by staining using the R.T.U Vectastain Universal Quick Kit (Vector Laboratories, Burlingame, CA). Finally, slides were counterstained with hematoxylin, dehydrated sequentially in ethanol and cleared with xylenes. The images were recorded by Leica slide scanner under a 10× or 40× objective (Leica). The intensity of the staining of PHLDA2/ p-AKT and p-ERK was scored by an expert pathologist (ACB) as 0 (no expression), 1 (weak expression), 2 (moderate expression) and 3 (strong expression).

### GFP and DsRed fusion constructs and Site-directed Mutagenesis

The GFP-PH-AKT construct was purchased from Addgene. We generated the following DsRed-PHLDA2 fusion constructs: Human wild-type PHLDA2 was cloned into the Bgl II/BamH I site of pDsRed-Express2-C1 vector (Clontech) with forward primer 5′-TGACCAAGATCTATGAAATCCCCCGACGAG-3′ and reverse primer 5′-TGACCAGGATCCTCATGGCGTGCGGGGTTTG-3′. Mutant PHLDA2 (amino acids 9–16 and 24–28 of human PHLDA2 were deleted) was generated with QuickChange Lightning Site-Directed Mutagenesis Kit (Agilent Technologies). Primers for the 9–16 a.a. deletion were: forward primer 5′-CCCCCGACGAGGTGCTAAGCGACAGC-3′ and reverse primer 5′-GCTGTCGCTTAGCACCTCGTCGGGGG-3′; primers for the 24–28 a.a. deletion were: forward primer 5′-CGACAGCCTCTTCCAGCTAGGGGTGCTCA-3′ and reverse primer 5′-TGAGCACCCCTAGCTGGAAGAGGCTGTCG-3′. Transient transfection assays were performed using X-tremeGENE 9 DNA transfection reagent (Roche).

### Immunofluorescence and confocal imaging

293T cells were maintained in DMEM medium containing 10% FBS with antibiotics. For confocal imaging of live cells, cells were plated onto 35 mm glass bottom dishes (MatTek Corporation) 24 hours prior to transfection. GFP-PH-AKT and/or DsRed-PHLDA2 constructs (0.5–2 μg of DNA) were transfected into these cells using X-tremeGENE 9 at a 1:3 DNA: X-tremeGENE 9 ratio (Roche Molecular Biochemicals) and were visualized directly at 24 hours post-transfection. The plate was then placed on the heated stage of a Zeiss Axiovert 100TV inverted fluorescence microscope (Microcosm Inc., Columbia, MD) with the temperature maintained at 37°C. Cells were photographed using a Nikon G4 microscope (Sterling, MA) with a SPOT RT (Kodak, Rochester, NY) camera for digital imaging.

### Expression and purification of Glutathione S-Transferase (GST) - fusion protein

GST-PH-AKT was generated as previously reported [28]. GST fusion constructs of PHLDA2 were prepared by PCR tagging of PHLDA2 cDNA with NdeI and BamHI sites at the 5′ and 3′ ends respectively, and subcloned into pGEX-2TL(+) vector (provided by Dr. Wenhui Zhao, Columbia University Medical Center, New York, NY). PCR primers were: upstream, 5′-TGACCACATATGAAATCCCCCGACGAG-3′ and downstream, 5′-TGACCAGGATCCTCATGGCGTGCGGGGTTTG-3′. The pGEX-2TL(+) constructs encoding PHLDA2 and the GST vector alone, were transformed into Rosetta™ 2 competent cells (Novagen), and a 1-liter culture was grown at 30°C in Luria broth containing 100 μg/ml ampicillin and 34 μg/ml chloramphenicol until the absorbance at 600 nm was 0.4–0.5. Isopropyl-β-D-thiogalactoside (100 μM) was added, and the cells were cultured for an additional 4 h at 30°C. The cells were pelleted and washed once with PBS at pH 7.4. The cells were resuspended in 25 ml of ice-cold BC500 buffer containing 20 mM Tris-HCl, pH 7.4, 500 mM NaCl, 0.2% Triton X-100, 10% Glycerol, 0.2 mM EDTA, 0.5 mM PMSF, and protease inhibitor mixture (Sigma), lysed by one round of freeze-thawing, and the lysates were sonicated on ice at power 3 (Sonic Dismembrator 550, Fisher Scientific, Pittsburgh, PA) to fragment the DNA. The lysates were centrifuged at 20, 000 × g for 30 min at 4°C, and the supernatant was then filtered through a 0.44-μm filter and incubated for 40 min on a rotating platform with 200 μl of glutathione-Sepharose pre-equilibrated in BC500 buffer. The suspension was centrifuged for 1 min at 3, 000 × g, and the beads were washed three times with 15 ml of BC500 Buffer and then two more times with 15 ml of BC100 buffer: 20 mM Tris-HCl, pH 7.4, 100 mM NaCl, 0.2% Triton X-100, 10% Glycerol, 0.2 mM EDTA, 0.5 mM PMSF, and protease inhibitor mixture. The protein was eluted from the resin at 4°C by incubation with 0.5 ml of BC100 buffer containing 20 mM glutathione. The eluate was divided into aliquots and stored at −80°C.

### Protein-lipid overlay assay

To assess the PIP binding properties of PHLDA2 protein, a protein-lipid overlay assay was performed using the GST fusion proteins and PIP Strips (PIP Strips™, Echelon). The strips were blocked in 3% (w/v) fatty acid-free BSA in TBST (50 mM Tris-HCl, 150 mM NaCl, 0.1% Tween 20, pH 8.0, 0.1% (v/v) Tween 20) for 1 h and were then incubated overnight at 4°C with gentle stirring in the same solution containing 500 ng/ml of the indicated GST fusion protein. The PIP strips were washed six times over 30 min in TBST and then incubated for 1 h with a 1:1000 dilution of anti-GST monoclonal antibody (Sigma). The strips were washed as before then incubated for 1 h with 1:5000 dilution of anti-mouse-horseradish peroxidase conjugate. Finally, the strips were washed 12 times over 1 h in TBST, and the GST fusion protein that was bound to the strips by virtue of its interaction with phospholipid was detected by Echelon's K-TMBP, which is a precipitation chromogen substrate that reacts with horseradish peroxide (HRP) producing a permanent purple colored spot. We then performed an *in vitro* protein-lipid overlay assay to see if PHLDA2 directly interferes with AKT binding to PIP. PI(3,4,5)P3 and PI(3,4)P2 were dissolved in a mixture of chloroform and methanol and H_2_O in 1:2:0.8, spotted onto nitrocellulose membranes (Bio-Rad), and air-dried. The membrane with phospholipids was blocked serially in 4% skim milk in TBST buffer and 3% fatty acid-free bovine serum albumin (Sigma) in TBST buffer for 30 min each at room temperature. GST-PHLDA2 and GST-AKT protein were mixed in a molar ratio of 1:1 and reacted with PI(3,4,5)P3 and PI (3,4) P2 lipids overnight at 4°C. After washing the membrane 6 times (10 min each) with TBST buffer, the membrane was incubated for 1 h at room temperature with 1:1000 dilution of anti-AKT PH domain antibody (Millipore). The membrane was washed as described above and then incubated with a 1:5000 dilution of HRP-conjugated secondary antibody. Finally, the membrane was washed with TBST buffer, and bound AKT was detected by K-TMBP (Echelon).

### Cell growth inhibition analysis

Cells were plated in each well of 96-well plates in cell medium containing 10% FBS. 24 hours after plating, cells were transfected with PHLDA2 siRNA and control siRNA for 48 hours and then treated with specified concentrations of inhibitors. The concentration of DMSO in the medium was adjusted to 0.1%. After 72 hours of incubation, viable cell numbers were measured through the use of 3-(4,5-dimethylthiazol-2-yl)-5-(3-carboxymethoxyphenyl)-2-(4-sulfophenyl)-2H-tetrazolium, inner salt (MTS; absorption of formazan at 490 nm; CellTiter96, Promega, Madison, WI) according to the manufacturer's protocol. Each assay consisted of three replicate wells of the same drug concentration. The IC50 was determined from the dose-response curves.

### Colony formation assay in soft agar

For anchorage independent growth assay, HCC827 cells were transfected with PHLDA2 siRNA and control siRNA for 48 hours and then trypsinized, counted twice using a hemocytometer, and plated at 5000 cells/ml in top plugs consisting of 0.4% SeaPlaque agarose (FMC Bioproducts, Rockland, ME) and RPMI 1640 medium. After 30 days, the colonies were photographed and counted. The experiment was repeated three times with two replicates each. Average numbers of colonies from each experiment were plotted.

### Statistical analysis

Statistical analysis was assessed by two-sample *t*-tests. The correlation between PHLDA2 and p-AKT, histopathological subtypes and EGFR status was performed using correlation *Z*-test in Statview version 5 (SAS Institute Inc. SAS Campus Drive, Cary, NC). A value of *P* < 0.05 was considered significant. The data are representative of three separate experiments (except for the immunohistochemistry study).
